# Brain iron redistribution in mesial temporal lobe epilepsy: a susceptibility-weighted magnetic resonance imaging study

**DOI:** 10.1186/s12868-014-0117-3

**Published:** 2014-11-21

**Authors:** Zhiqiang Zhang, Wei Liao, Boris Bernhardt, Zhengge Wang, Kangjian Sun, Fang Yang, Yijun Liu, Guangming Lu

**Affiliations:** Department of Medical Imaging, Jinling Hospital, Nanjing University School of Medicine, 305# Eastern Zhongshan Rd, Nanjing, 210002 China; Center for Cognition and Brain Disorders, Affiliated Hospital of Hangzhou Normal University, Hangzhou, 310015 China; Department of Social Neuroscience, Max Planck Institute for Human Cognitive and Brain Science, Leipzig, Germany; Department of Neurosurgery, Jinling Hospital, Nanjing University School of Medicine, Nanjing, 210002 China; Department of Neurology, Jinling Hospital, Nanjing University School of Medicine, Nanjing, 210002 China; Department of Psychiatry and Neuroscience, University of Florida, Gainesville, Fl USA

**Keywords:** Brain iron, Mesial temporal lobe epilepsy, Susceptibility-weighted magnetic resonance imaging

## Abstract

**Background:**

The roles of iron in epilepsy and its pathophysiological significance are poorly understood, especially whether iron levels are abnormal in subcortcal structures. This study aims to demonstrate whole-brain iron alterations and its clinical relevancies in mesial temporal lobe epilepsy (mTLE) *in vivo*, using susceptibility-weighted magnetic resonance imaging (SWI).

**Methods:**

We studied 62 patients with mTLE and 62 healthy controls. Brain iron concentration was quantified using SWI phase values. Voxel-wise analysis was carried out to compare iron levels between mTLE and controls, and to assess the relationship between altered iron concentration and clinical parameters in mTLE.

**Results:**

Patients with mTLE showed decreases of iron levels in the subcortical structures such as substantia nigra, red nucleus, and basal ganglia. Conversely, iron levels were decreased in the cortex. Subcortical iron levels were negatively correlated to those in the cortex. Moreover, cortical and basal ganglia iron levels were related to clinical variables including epilepsy duration, age at seizures onset, and histories of precipitating factors.

**Conclusions:**

Our SWI findings suggest a redistribution of iron between subcortical and cortical structures in mTLE. The degree of redistribution is affected by both progression of epilepsy and precipitating factors. Investigation on brain iron redistribution offers new insights into the pathogenesis of mTLE, and may be a potential biomarker for monitoring the clinical progression of epilepsy.

**Electronic supplementary material:**

The online version of this article (doi:10.1186/s12868-014-0117-3) contains supplementary material, which is available to authorized users.

## Background

Iron is essential for many brain physiological processes ranging from gene expression, neuronal development, enzymatic reactions, dopamine synthesis and electron transport [1-3]. Moreover, abnormal iron levels have been found in many neurological disorders, such as Parkinson’s disease [4], Alzheimer’s disease [5] and Restless Legs Syndrome [6], suggesting that the measurement of cerebral iron concentration may be a potential biomarker to advance the understanding, diagnosing, monitoring, and treatment of diseases [2,7].

There is initial evidence for a close link between alterations in brain iron and epilepsy [8-10]. In animal models, injection of ferric or ferrous chloride into neocortical regions or hippocampus has resulted in electrographic and behavioral seizures [9]. The free radicals generated by iron have been shown to attack cell membranes by lipid peroxidation, leading to neuronal damage and ultimately epileptic discharges [11]. In rat model of epilepsy, up-regulated expression of ferritin, an iron storage protein, has been observed in regions particularly vulnerable to cell death [10]. In human patients, observational data on a direct link between iron and epileptogenis is scarce. Increased iron burden, often subsequent to intraparenchymal hemorrhages and trauma, may be associated with an elevated incidence of epilepsy [12,13]. Other indirect evidence for a link between epilepsy and iron comes from work on resected tissue, where blood-brain barrier leakage, which may cause local extravasation of blood and release of iron from haemoglobin-containing blood cells, may take place secondary to seizures [14].

The invasive nature of histological approaches to measure iron levels [15] precludes their use in epileptic patients [16,17]. The recent development of magnetic resonance imaging (MRI) techniques based on susceptibility-weighted imaging (SWI), offers an accurate *in vivo* measurement of brain iron deposition [7]. Indeed, phase images of SWI, a high-resolution, 3D, and fully flow-compensated gradient echo sequence, are sensitive to subvoxel magnetic inhomogeneities affected by iron in the forms of haemosiderin, ferritin and deoxyhaemoglobin. It has been shown that SWI and can measure iron levels on the order of just 1 mg/g tissue in vivo [7,18]. While previous studies have used SWI to assess the iron alterations in non-blood brain tissue (non-heme iron) and their clinical relevance in Parkinson disease, Alzheimer Disease and multiple sclerosis [4,5,19]. There are to date no data on iron-related SWI changes in epilepsy.

The purpose of the current study was to employ voxel-based SWI analysis to investigate the topography of brain iron alterations in mTLE. (1) Given that the previous studies mostly focus alterations of iron concentrations in epilepsy in the cortical regions [8], the iron alterations in subcortical structures in epilepsy thus still remain poorly understood. In human brain, non-hemo iron is highly concentrated in the globus pallidus (GP), substantia nigra (SN) and red nucleus (RN), and is an essential cofactor in the synthesis of dopamine [20]. Considering the important roles of dopaminergic neurons and the subcortical structures played in mTLE [21,22], it is conceivable that the subcortical iron may be associated with the process of epilepsy. Hence we first assessed phase alterations in the subcortical structures in mTLE, and particularly interested in the relationship between subcortical and cortical phase alterations. (2) Considering that there is currently lack of clinical significances of iron alterations in epilepsy, we correlated brain phase values with clinical variables of epileptic patients, in order to explore the possible roles of brain iron played in the pathogenesis and progression in mTLE.

## Methods

### Participants

We recruited 62 consecutive adult patients with mTLE who had received clinical treatments in Jinling Hospital. Demographic and clinical data are detailed in the Table 1. MTLE diagnosis and lateralization of the seizure focus were determined by a comprehensive evaluation, including seizure history and semiology, neurological examination, diagnostic MRI, and EEG records in all patients. Patients were retrospectively selected if they satisfied the following criteria: (i) All patients were young and middle-aged adults. Patients younger than 18 yrs, or older than 50 yrs were excluded. (ii) Patients with other identifiable structural MRI abnormalities than hippocampal sclerosis, such as cortical dysplasia, vascular malformation or tumor were excluded. (iii) Pathogenesis of mTLE. Twenty-three patients with prior history of febrile convulsion, 14 patients with intracranial infections and 25 patients without pathogenic history were involved; patients with the other pathogenesis, such as head trauma and poisoning were excluded. Patients were compared to 62 age- and gender-matched controls, recruited from the staff of Jinling Hospital. Controls did not suffer from neurological or psychiatric disorders at the time of the study.

**Table 1:** **Demographic and clinical information of patients with mTLE and healthy controls**

**Groups**	**Men**	**Age**	**Duration**	**Onset age**	**Seizure frequency**	**History**	**Hippocampal sclerosis**
Controls (n = 62)	34	27.5 ± 8.1 (18-48)	None	None	None	None	None
LTLE (n = 31)	18	26.1 ± 7.6 (18-48)	11.8 ± 8.8 (1-29)	14.4 ± 8.9 (1-46)	3 (2-150)	FC: 13	LHS: 27
						II: 8	
						NH: 10	BHS: 4
RTLE (n = 31)	16	28.8 ± 8.4 (18-48)	10.5 ± 8.3 (1-38)	18.4 ± 9.8 (1-37)	5 (0.5-90)	FC: 10	RHS: 30
						II: 6	
						NH: 15	BHS: 1

LTLE: left temporal lobe epilepsy; RTLE: right temporal lobe epilepsy.

Age, duration of epilepsy and age at onset are presented in mean ± SD (range) years. Seizure frequency is given as median (range) of seizures/mo. II: intracranial infection; FC: febrile convulsion; NH: no prior history.

**Definitions:** *epilepsy duration*: period from the time of the first independent seizures to the time of MRI scan; *ages of seizures onset*, ages at the first independent seizures onset; and *seizure frequency*, seizures frequency over the recent one-year.

This study was approved by the Medical Ethics Committee in Jinling Hospital, Nanjing University School of Medicine (Reference number: 2012GJJ-055). All examinations were carried out under the guidance of the Declaration of Helsinki 1975. Written informed consent was obtained from all the participants.

### MRI data acquisition

MRI data were collected on a 3 T scanner (MAGNETOM Trio, Siemens Healthcare, Erlangen, Germany) equipped with an eight-channel, phase-array head coil. Whole-brain high-resolution SWI data including phase images were obtained parallel to the anterior-posterior commissural line using a 3-Dimensional gradient-echo sequence (TR/TE: 28/20 ms; flip angle: 15°; slices: 48; field of view: 240/240 mm; matrix: 448 × 358 and oversampling: 16.7%). SWI acquisition generated phase images, magnitude images, SWI with overlapping phase images on magnitude images and SWI with minimal intensity project (mIP) reconstruction [18]. These SWI images were all online processed automatically on a workstation using the Syngo VB17 software (Siemens Medical Solution), and phase images were high-pass filtered (64 × 64 low spatial frequency kernel). Moreover, we obtained high-resolution T1-weighted anatomical images using a 3D Magnetization Prepared Rapid Acquisition Gradient-echo (MPRAGE) sequence (TR/TE = 2300 ms/2.98 ms, FA = 9°, matrix = 256 × 256, FOV = 256 × 256 mm^2^, slice thickness = 1 mm). Moreover, coronal T1 (TR/TE = 280/2.5 ms; FOV = 230 × 230 mm^2^, slice thickness = 4 mm, no gap, 18 slices) and T2 FLAIR (TR/TE =8000/93 ms; FOV = 230 × 230 mm^2^, slice thickness = 4 mm, no gap, 18 slices) images were collected to measure hippocampal volume and detect hippocampal signal abnormalities.

### Data preprocessing

#### Intensity preprocessing of the phase images

Firstly, the unwarpped and high-pass filtered [7,18] phase images were linearly scaled to a phase value range of π to -π. Subsequently, voxels with positive phase value (i,e, those ranging between 0 and π) were removed. This step was performed to exclusively measure iron content in the brain, because it is well-known that iron in tissue has a negative-phase effect [18]. Secondly, we automatically identified brain veins showing dark signal intensity in the MIP images individually, and replaced the corresponding voxels in the phase images by mean global signal intensity of the phase image. Following these preprocessing steps, the phase values are believed to represent the content of the non-hemo iron deposition [18].

#### Spatial preprocessing of the phase images

**Figure 1:**
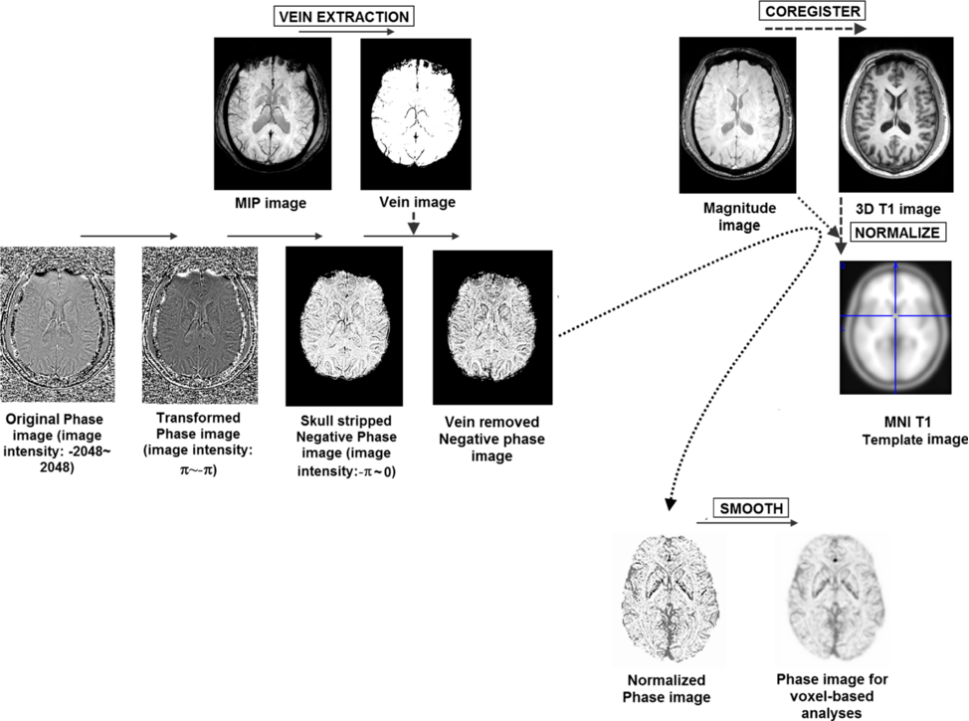
**Flow chart of data processing.**

Spatial preprocessing of the phase images was performed using SPM8 for Matlab. Data of patients with left-sided mTLE and controls were left-right flipped. Phase images of all subjects were linearly co-registered to their high-resolution T1-weighted images, then the affine matrix by normalizing the 3D anatomic images to a T1 template with Montreal Neurological Institute (MNI) 305 coordination was written to the phase images after intensity processing. Normalized phase images were further re-sampled to a resolution of 1.5 × 1.5 × 1.5 mm, and smoothed with a 4 mm Full-width-at-half-maximum Gaussian kernel to increase the signal-noise ratio (Figure 1). Prior to group analyses, subject-level voxel-wise phase value maps were standardized into subject-level *Z*-score maps (i.e., by subtracting the mean voxel-wise phase obtained for the entire brain, and then dividing by the standard deviation). The standardized phase can improve the subsequent statistical analyses on group-level phase measures [23].

### Voxel-based analyses of SWI phase values in mTLE

Phase images of all subjects were preprocessed for the removal of heme-iron confounds and spatially normalized to a standard stereotaxic space. Voxel-based analyses were carried out using SPM8 (http://www.fil.ion.ucl.ac.uk/spm) for Matlab (The Mathworks, Natick, MA).

#### Group comparison

We first observe the topological pattern of iron distribution in human brain by performing one-sample t-test on SWI phase values of all subject groups. We subsequently applied two-sample *t*-test on SWI phase values between patients and controls to map the topological pattern of altered brain iron in mTLE. The gray matter (GM) volume of each subject extracted from voxel-based morphometric analysis, was regressed voxel-wisely. Moreover, for validating the result of voxel-based analysis, we additionally performed ROI-based comparison analyses (See Additional file 1: Figure S1).

#### Correlation analysis between regions of iron changes

We performed a post-hoc correlation analysis to study the relationship between the subcortical showing altered phase values and rest of the brain. The bilateral RN and SN, which showed most intensively decreased phase values in patients with mTLE (see the Results for details), were combined and selected as seed region. A voxel-based correlation analysis measuring the covariance of phase values across subjects was conducted, which can detect the regions whose phase values were correlated with those in the seeds. The correlation patterns of the patients and controls were compared using a classic interaction linear model [24]. For each subject group, the phase values in the regions of RN + SN (seed region for correlation maps), cortex and basal ganglia were extracted out. The region of cortex and basal ganglia were defined according to the correlation map of the patients.

#### Subgroup assessment of effect of precipitating factors

Within the patient group, we applied a one-way analysis of variance (ANOVA) to compare patients with a prior history of febrile seizures (n = 23), patients with intracranial infections (n = 14), and patients with no overt history (n = 15). The ANOVA analysis was assumed to find the specific iron distribution patterns in mTLE with different precipitating factors.

#### Correlation analyses between the phase values and clinical variables in mTLE

Voxel-based correlation analyses were performed to highlight regions of correlation between SWI phase values and the clinical variables *epilepsy duration*, *age at seizures onset*, and *seizure frequency, (*as defined in Table 1). Because epilepsy duration was negatively correlated with age at seizure onset (*r* = -0.607, *p* < 0.001) in our patients, these two clinical variables were regarded as covariates each other in the voxel-based correlation analyses. During the above voxel-based analyses, subject age and gender were included in the model as nuisance covariates [25].

#### Correction for multiple comparisons

We used the false discovery rate (FDR, *P* < 0.05) procedure to correct for multiple comparisons during all voxel-based analyses [26].

## Results

### Patterns of abnormal SWI-phase in mTLE relative to controls

**Figure 2:**
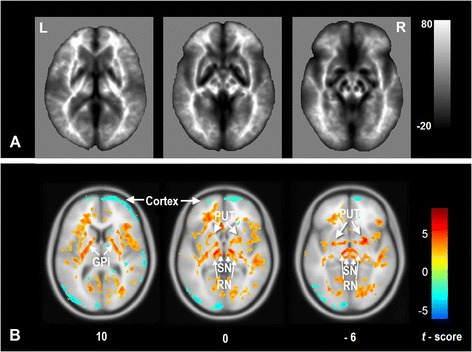
**Comparison of the whole brain phase values between the patients with mTLE and healthy controls. A**: One-sample t-test for processed phase images in all subjects. It is apparent that the sub-cortical nuclei, including the globus pallidus, substantia nigra and red nucleus showed lower phase values (or higher iron concentration) than the other brain structures. **B**: Two sample t-tests analyses revealed increased iron concentration in mTLE in the neocortex, and decreased iron concentration in the subcortical structures. SWI phase value were decreased in multiple cortical regions in mainly frontal, temporal and occipital lobes; increased SWI phase was found in sub-cortical structures including the bilateral GPi, RN and SN. Finding have been thresholded at *P* < 0.05, *FDR* correction. The results are shown by overlying on MNI 305 template image. *Abbreviations:* L: left; R: right; PUT: putamen; GPi: internal globus pallidus; SN: substantia nigra; RN: red nucleus.

One-sample t-tests revealed topological patterns of phase values. Apparently, the subcortical structures including the globus pallidus (GP), substantia nigra (SN) and red nucleus (RN) showed lower phase values in contrast to the white matter and cortex. Analyzing differences in global mean SWI phase, there was no statistical difference (*t* = 1.036, *p* = 0.328) between patients (*mean* ± *std*: -0.047 ± 0.009) and controls (-0.048 ± 0.009). On the other hand, voxel-wise group comparisons decreases in SWI phase (i.e., increased iron concentration) in mTLE in widespead cortical networks including frontal, temporal, and occipital cortical areas (*P* < 0.05, FDR correction), as well as increased phase (i.e., decreased iron concentration) in subcortical structures such as the bilateral internal globus pallidus (GPi), putamen (PUT), RN and SN (Figure 2). ROI-based comparison analyses repeated the VBA results (See Additional file 1: Table S1).

### Cortico-subcrotical iron level correlations

**Figure 3:**
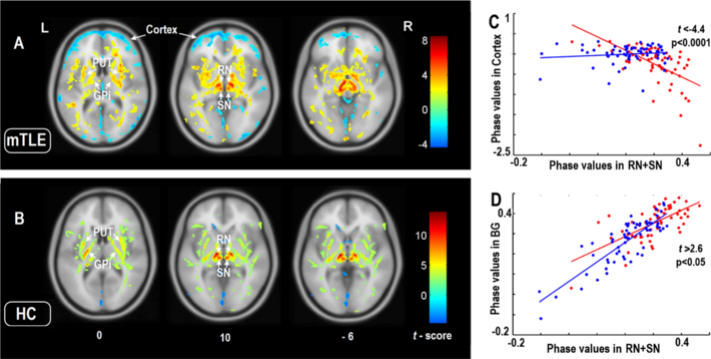
**Correlation analysis of cortical and subcortical iron levels.** Covariance analysis between cortical and subcortcial SWI phase values in patients with mTLE **(A)** and healthy controls **(B)**. Seeds were based on the peak coordinates of group difference in the SN and RN (see Figure 1). In mTLE, seed SWI phase was positively correlated to multiple subcortical regions, and negatively correlated with neocortical regions. In healthy controls, subcortical positive correlations were more restricted and there was no negative correlation between the seed regions and cortical areas. Findings have been thresholded at *P* < 0.05, *FDR* correction. The results are shown by overlying on MNI 305 template image.The right panels illustrate the differences of interregional correlation of phase values in patients and controls. **(C)**: Interregional correlations between the seed region (SN + RN) and the cortex. The phase values in the SN + RN was negatively correlated those in the cortex (r = -0.59, p = 4.92 × 10^-9^) in the patient group; but no significantly correlation was found in the control group (r = 0.12, p = 0.34). The correlations were different between the patients and controls (t = -4.44, p < 0.0001). **(D)**: Interregional correlations between the seed region (SN + RN) and the basal ganglia. The phase values in the SN + RN was positively correlated those in the basal gangila in both patient (r = 0.66, p = 4.92 × 10^-9^) and control groups (r = 0.85, p = 2.47 × 10^-18^). In the plots, phase values and regression lines are shown in red for the patients and in blue for controls. The correlations were different between the patients and controls (t = 2.61, p < 0.05). The comparisons of correlations were performed using a classic interaction linear model. *Abbreviations:* L: left; R: right; mTLE: mesial temporal lobe epilepsy; HC: healthy controls; PUT: putamen; GPi: internal globus pallidus; SN: substantia nigra; RN: red nucleus.

In mTLE, voxel-wise correlation analysis seeding from the RN and SN confirmed and extended the patterns seen in the group analysis. Indeed, we observed widespread positive correlations of RN/SN with the other basal ganglia such as PUT and GPi, and negative correlations with neocortical regions. This result indicates that patients with high iron levels in SN and RN also tend to have high iron levels in PUT and GP, but low iron in cortical areas. This negative correlation pattern was not found in controls (Figure 3 and Additional file 1: Table S2).

### Effect of precipitating factors

**Figure 4:**
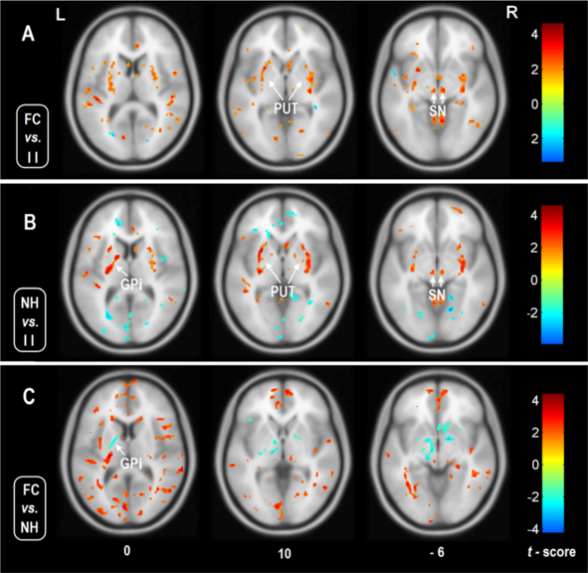
**Comparison of phase values among the patients with precipitating factors.** One-way ANOVA analysis revealed difference iron states between the patients with different pathogenesis (*p* < 0.05, FDR correction). Comparing with the subgroup of intracranial infection, the subgroups of febrile convulsion **(A)** and no overt history **(B)** both showed increased phase values, i.e., decreased iron concentration(warm color) in the PUT and SN. The difference of phase value between subgroups of FC and NH was located in the GPi **(C)**. The results are shown by overlying on MNI 305 template image. *Abbreviations:* L: left; R: right; mTLE: PUT: putamen; GPi: internal globus pallidus; SN: substantia nigra; RN: red nucleus; II: intracranial infection; FC: febrile convulsion; NH: no overt history.

We observed different patterns of iron deposition alterations in patients with different precipitating factors (Figure 4 and Table 2). Compared to the subgroup of intracranial infection, the subgroups of febrile seizures and no overt history both showed higher phase values (i.e., lower iron level) in the bilateral PUT, RN and SN. However, there were no significant alteration of phase values when comparing subgroup of febrile seizures and that of no overt history.

**Table 2:** **Results for group comparisons of phase values**

**Brain regions**		**mTLE** ***vs.*** **HC**	**FC** ***vs.*** **II**	**NH** ***vs.*** **II**	**FC** ***vs.*** **NH**
		x, y, z* ( ***t*** **)**	x, y, z* ( ***t*** **)**	x, y, z* ( ***t*** **)**	x, y, z* ( ***t*** **)**
Cortical structures	L Front	-18,44,15 (-3.22)	None	None	None
	R Front	38,51,18 (-4.98)	None	None	None
	L Temp	-44, 13, -10 (-3.63)	None	None	None
	R Temp	47, 0, 14 (-3.87)	None	None	None
	L Occip	-11, -94, 0 (-4.35)	None	None	None
Subcortical structures	L PUT	-23, 7, 2 (4.33)	-25, 9, 1 (3.55)	-25, 9, 2 (4.41)	None
	R PUT	21, 5, -2 (5.54)	31, -19, -2 (3.74)	30, -17, 4 (3.31)	None
	L GPi	-25, -16, 4 (6.53)	None	None	None
	R GPi	25, -13, 5 (6.21)	None	None	None
	L GPe	None	None	-15, 2, 11 (3.85)	-18, -4, 10 (-2.71)
	R GPe	None	None	None	19, -2, 10 (2.72)
	L SN	-5, -20, 0 (4.56)	-4, -19, -8 (2.98)	-4, -19, -9 (3.47)	None
	R SN	6, -21, 0 (4.47)	4, -20, 9 (3.45)	4, -20, -9 (3.22)	None
	L RN	-12, -7, -5 (5.92)	None	None	None
	R RN	11, -3, -3 ( 6.33)	None	None	None

*Abbreviations: mTLE* mesial temporal lobe epilepsy, *HC* healthy controls, *L* left, *R* right, *Front* frontal lobe, *Temp* temporal lobe, *Occip* occipital lobe, *PUT* putamen, *GPi* internal globus pallidus, *GPe* external globus pallidus, *SN* substantia nigra, *RN* red nucleus, *FC* febrile convulsion, *II* intracranial infections, *NH* no prior history of pathogenesis. Notes: *MNI coordinates.

### Effects of clinical variables

We observed a negative correlation between SWI phase in cortical regions and a majority of subcortical structures (including bilateral PUT, external globus pallidus [GPe], SN and RN) to duration of epilepsy. These findings indicate lower iron levels in patients with long-withstanding seizures in these regions. In the GPi, on the other hand, we observed a positive correlation, indicating progressive iron increase.

**Figure 5:**
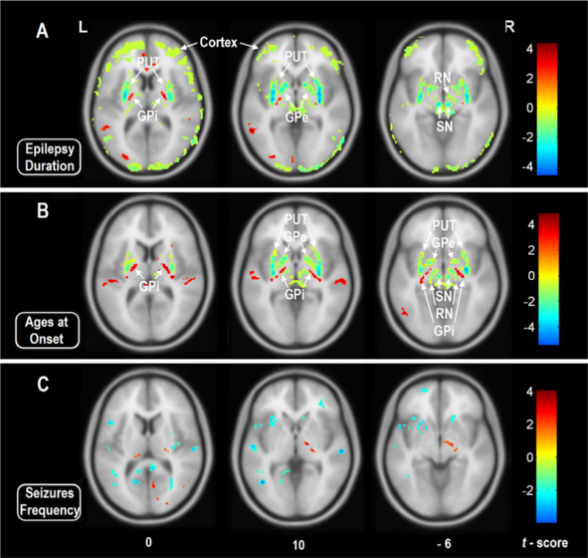
**Voxel-based correlation analyses between the phase values and clinical variables in the patients with mTLE.** Warm color denotes positive correlation, and cold color denotes negative correlation (*p* < 0.05, FDR correction). **A**: Correlation between phase values and epilepsy duration. The cortical and subcortical structures both showed negative correlation, denotes that the iron concentrations in these brain regions are positive correlated with epilepsy duration. **B**: Correlation between phase values and ages at seizures onset. Negative correlation was found between the subcortical structures including the GPe, PUT, SN and RN. In the results of A and B, the nucleus of GPi showed dissociated presence with the other subcortical nuclei. **C**: Correlation between phase values and seizures frequency. No meaningful region was found to have correlation. The results are shown by overlying on MNI 305 template image. *Abbreviations:* L: left; R: right; PUT: putamen; GPi: internal globus pallidus; GPe: external globus pallidus; SN: substantia nigra; RN: red nucleus.

Analyzing effects of seizure onset, subcortical finding resembled the pattern seen in the previous analysis of duration of epilepsy. Whereas, we did not observe any significant correlation in the cortical regions. We did not observe any correlation to seizure frequency in the brain regions as described above (Figure 5 and Table 3).

**Table 3:** **Correlation between phase values and clinical variables**

**Brain regions**		**Duration**	**Onset**	**Seizure frequency**
		x, y, z* ( ***t*** **)**	x, y, z* ( ***t*** **)**	x, y, z* ( ***t*** **)**
Cortical structures	L Front	-33, 62, 13 (-4.58 )	None	None
	R Front	48, 21, 23 (-4.04)	None	None
	L Temp	-36, 5, -30 (-4.07)	None	None
	R Temp	45, 6 -30 ( -3.23)	None	None
	L Occip	-16, -101, 13 (-4.47)	None	None
	R Occip	25, -101, 2 (-4.96)	None	None
Subcortical structures	L PUT	-30, -4, 1 (-6.42)	-28, -11, 4 (-6.72)	None
	R PUT	31, -4, 0 (-6.32)	30, -7, -5 (-7.62)	None
	L GPi	-21, -10, 4 (3.18)	-19, -8, 5 (3.10)	None
	R GPi	22, -8, 10 (3.46)	19, -7, 7 (3.19)	None
	L GPe	-13, 6, 1 (-5.53 )	-15, 6, 2 (-5.58)	None
	R GPe	-19, 2,15 (-3.93)	13, 6, 1 (-5.35)	None
	L SN	-12, -11, -3 (-2.72 )	-6, -17, -6 (-5.09)	None
	R SN	15, -13, 15 (-3.25)	7, -19, 9 (-5.92)	None
	L RN	-6, -19, -8 (-5.38)	-12, -11, -3 (-4.35)	None
	R RN	7, -19, -6 (-5.29)	13, -11, -5 (-4.73)	None

*Abbreviations:* *L* left, *R* right, *Front* frontal lobe, *Temp* temporal lobe, *Occip* occipital lobe, *PUT* putamen, *GPi* internal globus pallidus, *GPe* external globus pallidus, *SN* substantia nigra, *RN* red nucleus, *FC* febrile convulsion, *II* intracranial infections, *NH* no prior history of pathogenesis. Notes: *MNI coordinates.

## Discussion

Measuring the phase values of MRI-SWI, this study provided novel insights into the whole brain changes of iron state in patients with mTLE *in vivo*. We observed increased cortical brain iron deposition in the patients, together with decreases in the subcortical structures such as the PUT, GP, SN and RN. Strikingly, the negative correlation of phase values between the cortical and subcortical structures suggests that these iron alterations in the brain structures may result from redistribution of brain iron stores in epilepsy. Clinical analyses further implicate that the changes of cerebral iron deposition are relevant with, and may play important roles in the progression and pathogensis of mTLE.

### Pattern of altered brain iron distribution in mTLE

In cortical regions, we observed an increased cortical iron deposition in mTLE, which is consistent with the findings in previous animal and clinical research [10,12,14]. Moreover, our result for the first time provided the panorama of increased iron in the diffuse cortical structures. Increased levels of brain iron have previously been interpreted to be a causative factor of epilepsy due to the damaging effect of iron [8]. Increased iron can lead to lipid peroxidation and then affects protein function, which is associated with increased excitation (increased extra-cellular glutamate) and decreased inhibition (decreased function of GABA_A_ receptor) of neurons in epilepsy [1,3,6,8,27].

Conversely to the findings in the cortical regions, we observed decreased iron levels in the basal ganglia subcortical nuclei, including the bilateral PUT, GP, SN and RN in the patients. This novel finding revealed that these subcortical nuclei, which containing the highest levels of non-hemo iron in the brain, are sensitive to the disturbance of iron metabolism in epilepsy. Basal ganglia subcortical nuclei may contribute to the patho-physiological process of epilepsy through their role in unilateral dystonic posturing seizure and inhibition of seizure activity [21,28,29]. SN and RN play important roles in propagation of seizure activity [22]. Given that the subcortical structures are densely innervated by dopaminergic pathways, and iron is an essential cofactor in the synthesis of dopamine [20], our findings may also be seen as suggestive of a link between epilepsy and dopamine insufficiency. A previous ^18^ F-Fluoro-L-DOPA PET study has found marked decreases in ^18^ F-Fluoro-L-DOPA uptake in the SN and PUT, suggesting impairment of dopamine activity in the patients with mTLE [22]. In disorders associated with subcortical iron deficiency, such as restless legs syndrome and Attention-Deficit/Hyperactivity Disorder, decreased iron in the subcortical nuclei have been shown to relate to alterations in dopamine production [30,31]. Moreover, in degenerative disorders, such as Parkinson’s disease and Alzeimer’s disease, increased subcortical iron levels have also been correlated with abnormal dopamine function [3-5]. Future study is needed to directly link MRI phase changes with dopamine levels in epilepsy.

Our correlation analysis showed that iron alterations in cortical structures in mTLE were negatively correlated with those in subcortical structures. These findings thus provide further quantitative evidence that higher cortical iron relates to lower subcortical iron, suggesting a coupled subcortico-cortical iron redistribution in mTLE. A similar pattern has been reported in a previous SWI study on laminar necrosis with hypoxia, a finding interpreted as iron being transported from ganglia to cortical structures [32]. Indeed, iron is taken up by the capillary endothelial cells from the circulation via transferrin receptors and gets sequestered in the basal ganglia [33]. The iron can also be transported from the basal ganglia along the axons to their sites of projection, where it may accumulate [33,34]. Although future studies are needed to provide more details on the exact directionality underlying our findings in epilepsy, increased cortical iron levels in mTLE may thus, at least in part, endogenously stem from subcortical structures.

### Alterations of brain iron and clinical relevancies

This study investigated the relationship of several clinical factors to altered iron levels in mTLE. Although the previous basic researches have correlated the excessive iron to the cause [8] or consequence [10,14] of epilepsy, our study provided clinical evidence for interrogation of relationship between iron and epilepsy. Firstly, the negative correlation between the epilepsy duration and phase values indicated that the altered brain iron is related to the progression of epilepsy. Moreover, the negative correlation between the ages at seizure onset and phase values in the subcortical nuclei may indicate that the iron level in epileptic brain may be affected by the ages of seizure onset.

Subgroup comparing results among the patients suggested that the brain iron alterations might also be associated with the pathogenesis of mTLE. Patients suffering from intracranial infections showed relative high iron concentration in the PUT and RN in contrast to the other two subgroups. It is conceivable that the alteration of iron levels in intracranial infections may arise from iron-accumulation by immune cells, e.g., microglial and astrocytes are known to uptake iron more readily than neurons [10,35].

### Methodological consideration and limitations

SWI is a mature and widely used MRI technique that can examine the brain iron either in the form of heme iron, i.e., deoxyhaemoglobin, or non-heme iron, such as ferritin and haemosiderin [7,18]. Only non-heme iron is considered to be directly relevant to the iron metabolism [7]. Many studies have demonstrated the correlation between the SWI phase shifts and brain iron concentrations in the human brain tissue [4,7]. In this work, we made a few of improvements to data analysis. Firstly, we applied voxel-based analysis for phase images instead of traditional manual ROI based analysis, which can detect the statistically significant results over the whole brain with independence of any prior hypotheses. Secondly, based on a vessel segmentation, we removed components of positive phase related to veins prior to statistical analysis, a step that likely eliminated confounds related to calcium and hemodeoxyhaemoglobin [7]. In addition, although we performed a voxel-wise gray-matter volume regression in statistical analysis, the morphological effect on phase data might not be thoroughly excluded in the cortical structures. Whereas, the selective appearances of cortical changes in MRI susceptibility might indicate a pathological phenotype in epilepsy, instead of the artifact effect from cortical atrophy.

Nevertheless, this study has several limitations. Firstly, because of a diverse antiepileptic drug treatment in our patients, we could not evaluate the possible effects of antiepileptic drugs on iron alterations in mTLE [36]. Secondly, a future follow-up study is required to further clarify the relationship between the iron state and treatment outcomes, in particular with respect to seizure-freedom following surgery. Thirdly, although no patient with anaemia was found, no available data of plasma ferritin levels might be the defect of this study. Previous evidence has suggested that the status of plasma ferritin may be correlated with seizures [37,38]. Fourth, data of left-sided mTLE and matched controls were left-right flipped to produce a homogenous right-sided mTLE dataset. While this approach aimed at increasing statistical power in comparing patients to controls, it may have introduced some confounds with respect to physiological lateralization of iron deposition [25]. Finally, with the improvement of MRI technique, the most newly-developed quantitative susceptibility mapping is more accurate than SWI on assessment of brain iron content [39].

## Conclusion

By measuring the phase values of the brain using susceptibility-weighted MRI, this study revealed a whole-brain pattern of iron alterations in mTLE. Besides to the anticipated finding that the cortical structures showed increased iron, we provide novel evidence showing decreased iron in the subcortical regions in mTLE. The negative correlation of iron concentrations in these two areas might suggest iron redistribution in epileptic brain. Clinical correlation analyses indicated that iron alterations are not only related to the clinical progression, but also to the precipitating factors. This study shed light on the pathophysiological mechanisms of iron alteration in mTLE, and provided a potential biomarker for studying epilepsy.

## Additional file

Additional file 1: Figure S1. Example of ROIs selections. Four ROIs, including the frontal cortex (purple), GPi (green), RN (red) and SN (white) ipsilateral to the epileptogenic side, were selected according to the VBA results. Especially, the ROI drawing of the frontal cortex was carefully performed to deliberately avoid the artifact at the interface between cortex and the skull. The phase values in each ROI (drawn by 3 operators, the values were averaged) were extracted from the patients and controls. The values were compared between the patient and control groups using two-sample t tests (Table S1). **Table S1.** Phase values in the ROIs. **Table S2.** Across-subject correlation analyses of phase values seeding at the RN+SN.
