# Supplementary material for: Brain iron redistribution in mesial temporal lobe epilepsy: a susceptibility-weighted magnetic resonance imaging study
Source: BMC Neurosci. 2014 Nov 21;15:117. doi: 10.1186/s12868-014-0117-3 (PMC4243317; doi:10.1186/s12868-014-0117-3)
Supplement: Additional file 1: Figure S1. — Example of ROIs selections. Four ROIs, including the frontal cortex (purple), GPi (green), RN (red) and SN (white) ipsilateral to the epileptogenic side, were selected according to the VBA results. Especially, the ROI drawing of the frontal cortex was carefully performed to deliberately avoid the artifact at the interface between cortex and the skull. The phase values in each ROI (drawn by 3 operators, the values were averaged) were extracted from the patients and controls. The values were compared between the patient and control groups using two-sample t tests (Table S1). Table S1. Phase values in the ROIs. Table S2. Across-subject correlation analyses of phase values seeding at the RN+SN. [file 12868_2014_117_MOESM1_ESM.doc]

**Supplementary Materials for**

**Brain Iron Redistribution in Mesial Temporal Lobe Epilepsy**

Zhiqiang Zhang,^1^ Wei Liao,^2^ Boris Bernhardt^3^, Zhengge Wang,^1^ Kangjian Sun, ^4^ Guanghui Chen, ^5^ Yijun Liu,^6^ Guangming Lu,^1*^

*1. Department of Medical Imaging, Jinling Hospital, Nanjing University School of Medicine, Nanjing 210002, China.*

*2. Center for Cognition and Brain Disorders, Affiliated Hospital of Hangzhou Normal University, Hangzhou 310015, China.*

*3. Department of Social Neuroscience, Max Planck Institute for Human Cognitive and Brain Science, Leipzig, Germany.*

*4. Department of Neurosurgery, Jinling Hospital, Nanjing University School of Medicine, Nanjing 210002, China.*

*5. Department of Neurology, Jinling Hospital, Nanjing University School of Medicine, Nanjing 210002, China.*

*6. Department of Psychiatry and Neuroscience, University of Florida, Gainesville, Florida, USA.*

**Corresponding author:**  Zhiqiang Zhang **or** Guangming Lu, MD

**Corresponding author’s address:** Department of Medical Imaging, Jinling Hospital, 305# Eastern Zhongshan Rd. Nanjing, 210002, China.

**Corresponding author’s phone and fax:** 86-25-80860185 (Tel); 86-25-84804659 (Fax).

**Corresponding author’s e-mail address:** zhangzq2001@126.com **or** [cjr.luguangming@vip.163.com](mailto:cjr.luguangming@vip.163.com)

**Running head:** (limit 50 characters): *Brain Iron Redistribution in mTLE*

**SUPPLMENTARY MATERIALS:**

***Supplementary Material 1. ROI based comparisons of phase values***


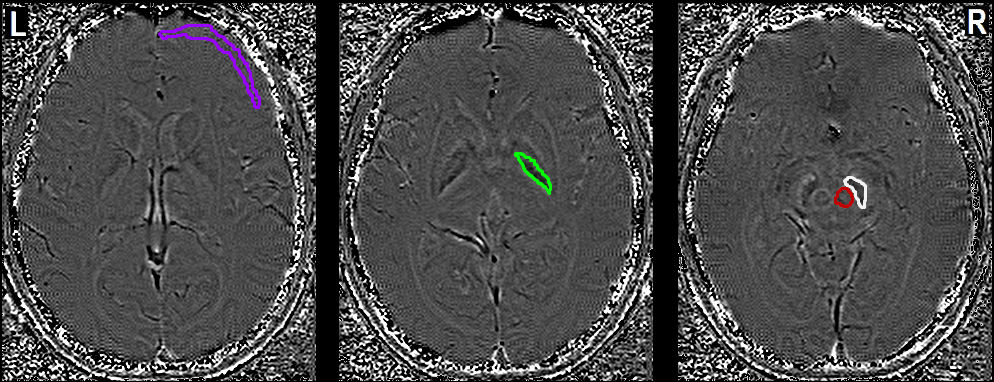


**sFigure 1. Example of ROIs selections**

Four ROIs, including the frontal cortex (purple), GPi (green), RN (red) and SN (white) ipsilateral to the epileptogenic side, were selected according to the VBA results. Especially, the ROI drawing of the frontal cortex was carefully performed to deliberately avoid the artifact at the interface between cortex and the skull. The phase values in each ROI (drawn by 3 operators, the values were averaged) were extracted from the patients and controls. The values were compared between the patient and control groups using two-sample t tests (s Table 1).

**sTable 1**. Phase values in the ROIs

| ROIs | Phase values in mTLE  (Mean±std) | Phase values in HC  (Mean±std) | Two sample t-tests  (Two-tailed) |
| --- | --- | --- | --- |
| r Front | -0.0333±0.0013 | -0.0186±0.0012 | *t*=6.602, *p*<10^-4^ |
| r GPi | -0.0365±0.0010 | -0.0468±0.0011 | *t*=6.252, *p*<10^-4^ |
| r RN | -0.0289±0.0009 | -0.0359±0.0010 | *t*=4.005, *p*<10^-4^ |
| r SN | -0.0344±0.0008 | -0.0433±0.0012 | *t*=5.765, *p*<10^-4^ |

*Abbreviations:* mTLE: mesial temporal lobe epilepsy; HC: healthy controls; PUT: putamen; GPi: internal globus pallidus; SN: substantia nigra; RN: red nucleus.

***Supplementary Material 2. sTable 2.*** ***Across-subject correlation analyses of phase values seeding at the RN+SN***

| Brain regions | | mTLE (L, R) | HC |
| --- | --- | --- | --- |
| Cortical  structures | Frontal | -3.47; -3.49 | none |
|  | Temporal | -2.75; -2.70 | none |
|  | Parietal | -2.49; -2.91 | none |
|  | Occipital | none; -3.10 | none |
|  |  |  |  |
| Subcortical  structures | PUT | 4.35; 5.93 | 6.42; 5.09 |
|  | GPi | 4.03; 5.29 | none |
|  | GPe | 2.44; 3.89 | 8.47; 7.85 |

*Abbreviations*: mTLE: mesial temporal lobe epilepsy; HC: healthy controls; PUT: putamen; GPi: internal globus pallidus; GPe: external globus pallidus; SN: substantia nigra; RN: red nucleus.
